# Drought driven shrinkage of surface water bodies in India

**DOI:** 10.1016/j.isci.2026.116737

**Published:** 2026-07-13

**Authors:** M. Niranjannaik, Vimal Mishra

**Affiliations:** 1 Earth Sciences, Indian Institute of Technology (IIT), Gandhinagar, Gujarat, India; 2 Civil Engineering, Indian Institute of Technology (IIT), Gandhinagar, Gujarat, India

**Keywords:** surface water area, trend, drought severity, shrinkage rate

## Abstract

Surface water bodies (SWBs) are vital in sustaining regional water storage, ecosystems, and water demands. However, the impact of climate extremes, particularly droughts, on these SWBs in India remains poorly understood. Here, we examine the changes and variability of the 10,476 SWB during 1990–2017 based on the Landsat satellite-derived SWB area, using Trend-Free Pre-Whitening Mann-Kendall (FPW-MK) and Sen’s slope tests. We also evaluated the impacts of drought severity (defined using Standardized Precipitation-Evapotranspiration Index) on SWB. We found that 79% of SWB across India exhibit a significant (*p* < 0.05) trend, with 44.5% decreasing and 29.4% increasing. Seasonal area anomalies reveal that a dry summer-monsoon causes the highest decline (5%) in SWB, compared to dry winter (2.7%) and dry pre-monsoon seasons (0.65%). Moreover, successive droughts during the summer-monsoon and winter seasons result in 3-fold more shrinkage (∼10%) than a single-season drought. Our findings highlight the critical need for strategies to sustainably manage SWB across India.

## Highlights

• Drought severity strongly controls SWB dynamics across India’s climate zones
• Successive seasonal droughts cause more SWB shrinkage than single-season droughts
• Arid and semi-arid SWB are most drought-prone, while mountain SWB are expanding
• Small and medium SWB are more vulnerable to drought than large SWB

## Graphical abstract

**Figure:**
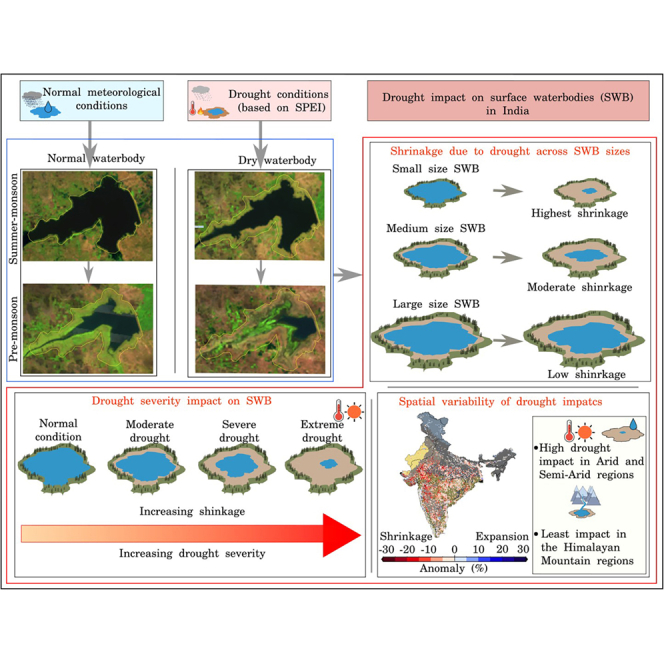

## Teaser

Environmental analysis; Environmental monitoring; Environmental science

## Introduction

Surface water bodies (SWBs), including lakes, ponds, and reservoirs, are crucial hydrosphere components.^1^ Even though their small proportion (20% of surface freshwater and 0.5% of total fresh water) on the Earth, these play a disproportionately important role in the hydrological cycle,^2^^,^^3^ by acting as a source of storage, buffer in the hydrological cycle, regulation in surface runoff, and redistribution of water around the globe.^4^ Additionally, SWB provide freshwater for drinking, agriculture, and industrial purposes. SWB offer valuable services to biodiversity and aquatic ecosystems, and maintain the ecological sustainability of the surrounding environment and human well-being.^5^ However, SWB are becoming increasingly vulnerable to climate extremes, particularly droughts, disrupting the balance between inflow and outflow.^6^ During droughts, a reduction in precipitation and increasing evapotranspiration causes a decline in water level and shrinkage of surface area, and in some cases, leading to the complete drying of SWB.^7^ Droughts threaten water availability in the SWB, which may affect food security and alter ecosystem functioning.^8^

Globally, there is growing evidence of significant shrinkage and water stress in SWB linked to prolonged drought events, increasing temperatures under climate change, and unsustainable water use by anthropogenic activities.^9^^,^^10^^,^^11^ SWB have witnessed a considerable decline in water levels, particularly in the regions of Central Asia, Africa, Australia, and parts of North America and Europe, where recurrent droughts led to long-term reductions in volume and surface area.^12^^,^^13^^,^^14^^,^^15^^,^^16^ Conversely, glacial lakes around the globe have experienced rapid growth in their size and numbers in response to climate change and the melting of the glaciers.^17^ For instance, glacial lakes in the Himalayan region are rapidly expanding their area and new lake formations, posing a severe threat of glacial outburst floods in the downstream regions.^18^^,^^19^

**Figure 1:**
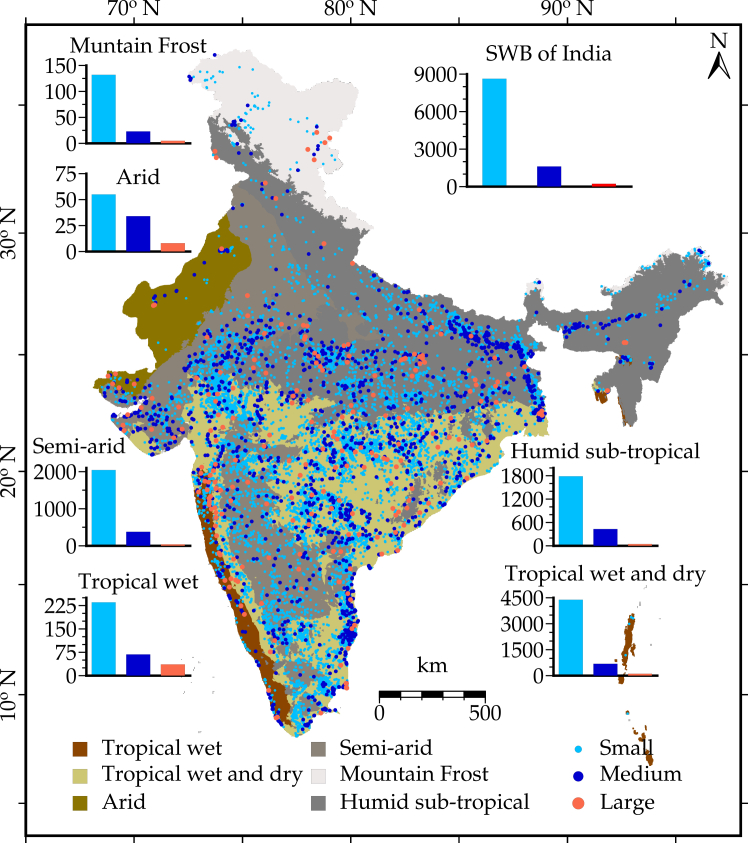
The spatial distribution of surface water bodies (SWBs) in India The SWBs are categorized into small (0.1–1 km^2^), medium (1–10 km^2^), and large (>10 km^2^) sizes, overlaid on the Köppen-Geiger climate zone classification map. The small, medium, and large SWBs are shown as light blue, dark blue, and red circles, respectively. The histograms inside the map show the number of SWBs in size categories of small, medium, and large SWBs within the respective climate zones.

India has a vast number of SWB (lakes, ponds, and reservoirs) that play a crucial role in water storage, agriculture, and provide freshwater sources for drinking purposes.^20^ These SWB are further categorized into small (0.1–1 km^2^), medium (1–10 km^2^), and large (>10 km^2^) sized SWB based on the previous studies.^13^^,^^21^ The SWB of varying sizes (small, medium, and large) play a critical role in regional water availability and climate interaction.^22^ Their distribution across different climate zones highlights the spatial variability in surface water resources availability (Figure 1). These SWB are under considerable water stress due to the high water demand,^23^ dense population,^24^ and increased climate extremes including droughts and heat waves.^25^^,^^26^^,^^27^ Additionally, SWB are heavily dependent on the summer-monsoon precipitation, which makes them even more vulnerable.^28^ Diverse climatic regions in India range from Arid to Tropical, making SWB highly sensitive to seasonal and interannual climatic variability.^29^ The frequency and severity of these extreme climate events are projected to increase in the future, which poses a severe threat to water and food security in India.^30^^,^^31^ Therefore, it is essential to understand the impacts of drought on SWB for effective water management, considering climate change.

Although variability in the area of SWB has been extensively studied,^29^^,^^32^ the impacts of severe droughts on SWB across different climate zones and seasons remain largely unexplored in India. To address these research gaps, we formulated three key research questions: (1) What are the impacts of droughts on SWB in India? (2) How does increasing drought severity from moderate to extreme affect SWB? (3) How do the shrinkage patterns of SWB vary during droughts in India? Understanding the drought impacts on SWB in India can provide crucial insights for water resource planning, climate adaptation, and ecosystem management.

## Results

### Changes in surface water bodies in India

**Figure 2:**
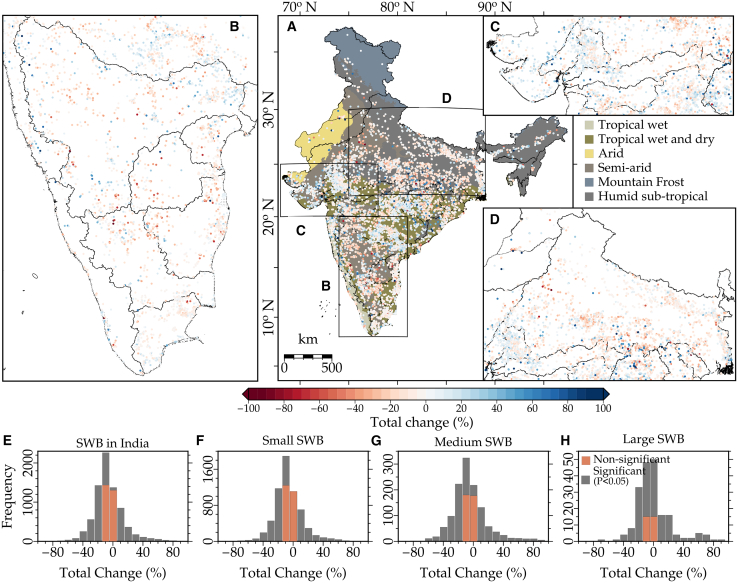
The long-term (1990–2017) trends of the surface water bodies (SWBs) area The long-term trends are expressed as total change (%) in the SWBs area for the 1990–2017 period, estimated from the trend slope (Equation 1) using the Trend-Free Pre-Whitening Mann-Kendall (TFPW-MK) method. The total percentage change in the SWB, with significant (*p* value <0.05) increasing and declining trends, is represented by blue and red circles, respectively. (A) The total change (%) in SWB water area, with a significant trend overlaid on the climate zones and river basins of India. (B) Subplot of the SWB with total change (%) of the South Indian Krishna, Kauveri, and Pennar river basins. (C) Subplot of the SWB total change (%) of the westward-flowing Narmada, Sabarmati, Mahi, and River Basins. (D) The total change (%) of SWB in the Ganga Basin. (E-H) represents the distribution of total change (%) of SWB with significant trends (gray bars) and non-significant (light red bars) for all SWB of India, and size-based categories such as small (0.1–1 km^2^), medium (1–10 km^2^), and large (>10 km^2^) SWB, respectively. For the percentage of SWB with a significant increasing and declining trend at the subbasin scale, see Figure S1.

We estimated non-parametric trends in SWB area from 1990 to 2017 over 10476 SWB and converted them into the total percentage change in SWB area (Equation 1). We find that 7739 (73.9%) SWB have experienced a significant (*p* < 0.05) trend (Figures 2A–2D). Among these, 4657 (44.5%) SWB faced a significant decreasing trend, whereas 3080 (29.4%) SWB showed a significant increasing trend (Figures 2E–2H). The long-term trends in the SWB area show a pronounced, significant decline in several subbasins across the north and south Indian regions (Figures 2 and S1). Approximately 80% of SWBs in the Ghaggar subbasin within the semi-arid zone exhibit a significant decreasing trend (Figures S1B and S1D). Widespread declines are also evident in the Ganga Basin, where 60%–80% of SWBs show significant reductions in the Gandak, Sone, upper Gomti, and upper Yamuna subbasins under a humid subtropical climate (Figures 2D, S1B, S1D). Additionally, 40%–60% of SWBs in the Krishna, Pennar, and Kaveri subbasins of the semi-arid zone display significant decreasing trends (Figure 2B). In contrast, more than 80% of SWBs in the Mountain Frost climate zone exhibit a significant increasing trend, likely driven by enhanced glacial meltwater contributions due to rising temperatures (Figure 2A). Similarly, more than 40% of SWBs show a significant increasing trend in the westward-flowing lower Narmada and Bhadar subbasins (Figures S1A and S1C). Moderate increase (40%–60%) in SWB is observed across some of the west-flowing river subbasins (Figure S1A), including the Narmada, Sabarmati, Mahi, Luni, and Shetranjali, as well as the east-flowing lower Godavari sub-basin, which is predominantly located in semi-arid climate zones. These increases are consistent with recent precipitation intensification over the region. Predominantly, small-sized SWB shows a significant declining trend in all the climate zones except the mountain frost zone, while the large SWB shows a significant increasing trend across all the climate zones. The majority of the medium-sized SWB shows a significant declining trend in arid and humid subtropical zones, but a significant increasing trend in tropical wet, tropical wet and dry, and semi-arid regions.

Overall, the area of SWB in the Humid sub-tropical climate zone has declined with a median change of −14.3%. However, a few SWB in the Semi-arid climate zones of South India have shrunk by more than 50% (Figure 2). In contrast, most of the SWB in the Semi-arid and Tropical wet and dry climate zones of central India shows an increasing trend in water area.

### Variability in the surface water area during dry and wet periods

**Figure 3:**
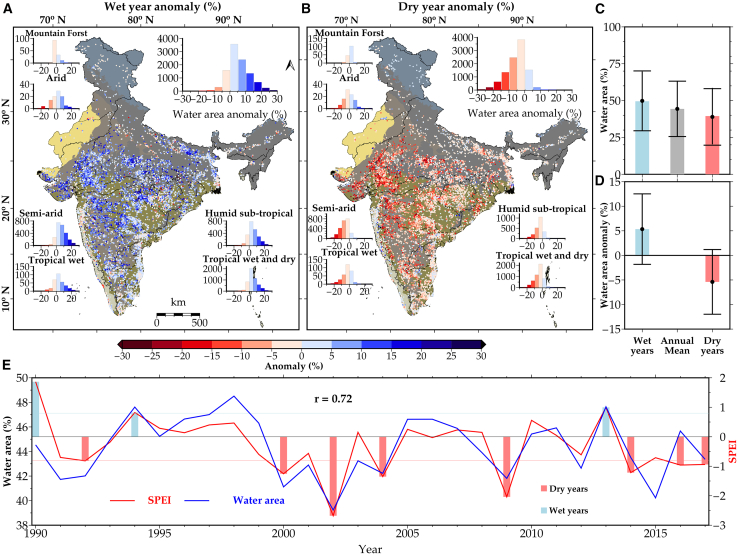
The spatial map of surface water bodies (SWBs) and the mean annual water area anomaly (in %) (A) wet year (SPEI > 0.8) water area anomaly and (B) dry year (SPEI < −0.8) water area anomaly, in respective subbasins of SWB, and the histograms show the count of SWB within the 5% range of mean water area anomaly for each climate zone. The blue circles and barrs indicate positives anomalies, while red circles and bars indicate negetive anomalies. (C) The mean annual water area (in %) of all the SWB in India during wet years, long-term mean, and dry years, respectively. (D) The mean annual water area anomaly (in %) of all the SWB in India during wet years, long-term mean, and dry years, respectively. The error bars represent the interquartile range. (E) The comparison of 12-month SPEI over the Indian region and the mean annual water area of all SWB in India. The identified wet and dry periods are highlighted with light blue and light red bars.

We estimated the anomaly (deviation from long-term mean) of mean annual SWB area (%) during wet (SPEI > 0.8) and dry (SPEI < −0.8) years from 1990 to 2017 across India (Figure 3). During the wet years, the majority of SWB (80%) experienced an expansion of water area, indicating a strong response to favorable hydroclimatic conditions (Figure 3A). In contrast, a small fraction of SWB showed minimal contraction during the wet years. Our findings show that the SWB in the tropical wet and dry and semi-arid climate zones gain water area during wet years. In contrast, 67% of SWB in the Mountain Frost climate zone shows shrinkage in water area (15.7 km^2^). This shrinkage in water area is observed despite an increase in precipitation (250 mm/yr) during wet years, likely due to a 1°C decline in mean annual temperature, causing shrinkage in SWB in the Mountain Frost region, possibly due to frozen SWB.^33^

During dry years, most SWB (82%) exhibit shrinkage (Figure 3B), highlighting the widespread sensitivity of surface water extent to drought conditions. The shrinkage is evident across all climate zones, except for the Mountain Frost climate zone, where the majority of SWB showed expansion. The SWB in semi-arid climate zones experience the most pronounced shrinkage (20%), reflecting their strong dependence on precipitation. In contrast, the expansion of SWB in the Mountain Frost climate zone is attributed to the warmer temperatures, which melt the glaciers and snow, resulting increase in water availability.^34^ The mean annual water area shows a strong positive correlation (r = 0.72) with the 12-month SPEI, indicating that drought conditions at the annual scale are closely linked to changes in SWB water extent. Our observations show that the major droughts (i.e., 2002 and 2009) and wet events (i.e., 1994 and 2013) in India coincide with the periods of widespread SWB shrinkage and expansion, respectively (Figure 3E). Therefore, assessing drought impact on water area at an annual scale is more suitable than at seasonal and monthly time scales.

**Figure 4:**
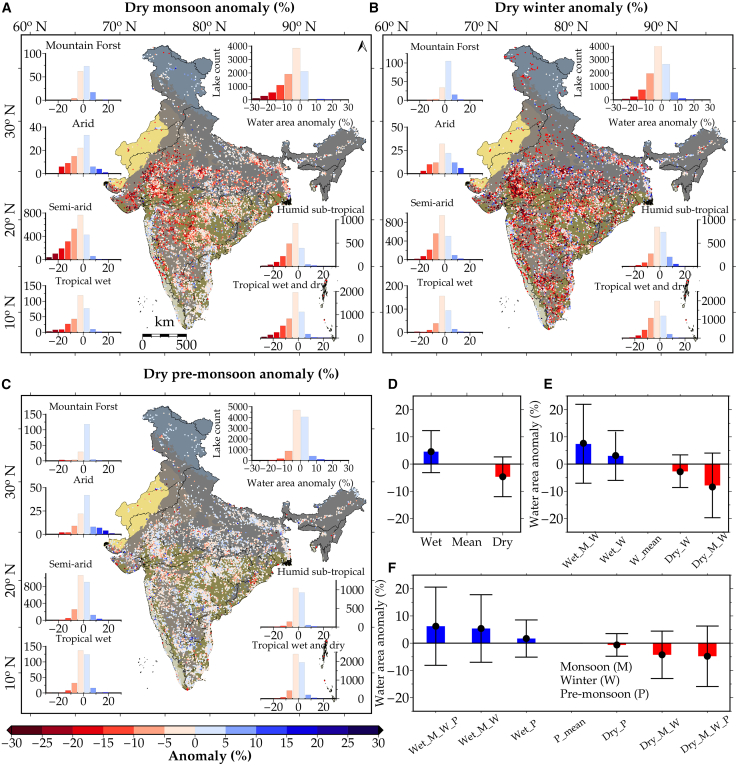
The spatial distribution of surface water bodies (SWBs) area anomaly during the dry seasons (A–C) Spatial maps show the mean water area anomaly during the summer-monsoon season, (B) winter, and (C) pre-monsoon seasons, respectively. (D–F) Bar plots of the mean water area anomaly of wet and dry periods for individual and combined seasons during the monsoon, winter, and pre-monsoon seasons, respectively. The blue and red bars indicate the wet and dry-period anomalies, respectively. The error bars represent the interquartile range. For the wet-season SWB anomaly, see Figure S2.

Furthermore, we examined the variability in the SWB area during the summer-monsoon (Figures 4A and 4D), winter (Figures 4B and 4E), and pre-monsoon (Figures 4C and 4F) seasons to assess the impact of droughts on their extent. Similarly, wet season impacts are also assessed on SWB variability (Figure S2). Seasonal analysis reveals that drought impacts on SWB vary strongly by season (Figure 4). The summer-monsoon season emerges as the most critical period, as the SWB are primarily dependent on monsoon precipitation. During the dry summer-monsoon season, the majority of the SWB in the semi-arid, humid subtropical, and tropical wet and dry climate zones of central India exhibit high shrinkage (>10%) (Figure 4A). Conversely, the SWB in the Mountain Frost and Arid climate zones are the least affected. The water area anomalies for the wet and dry summer-monsoon seasons are estimated as 4.55 ± 7% (928.65 km^2^) and −4.67 ± 7% (953.15 km^2^) (Figure 4D).

Subsequently, winter droughts led to a 2.5% shrinkage in India, particularly SWB, in the Semi-arid and Tropical wet and dry climate zones of the central Indian region (Figure 4B). Importantly, successive dry seasons within a single water year result in the highest water loss. The SWB experiencing consecutive dry summer-monsoon and winter seasons of the water cycle shows an 8.5% water area shrinkage, which reveals that the SWB area experienced nearly three times greater shrinkage than those affected by a single summer-monsoon dry season. The Wilcoxon rank-sum test shows significantly greater SWB shrinkage during successive droughts than single-season droughts in both seasons, with median differences of 4.35% (monsoon) and 5.25% (winter) (see Table S1). The successive seasonal shrinkage demonstrates a strong compound effect of consecutive dry seasons on the SWB area (Figure 4E). The seasonal SWB area anomaly indicates that the dry monsoon alone causes a 5% decline in the mean SWB area in India. Whereas the dry winter and pre-monsoon seasons resulted in a decline of 2.7% and 0.65%, respectively. The dry monsoon season has a greater impact, even when other seasons are wet, resulting in a 1.33% reduction in winter. This indicates that a dry monsoon season, even when followed by wet seasons, is unlikely to restore its long-term mean SWB area. The SWB experienced shrinkage in water area across all the climate zones, except the Mountain Frost zone, with the most severe shrinkage observed in the Semi-arid and Tropical Wet and Dry zones. The SWB water area anomalies during the dry winter highlight exacerbated surface water loss, particularly in the Narmada, Sabarmati, Mahi basins of central India and Godavari, Kaveri, and Krishna Basins of the southern peninsular regions, due to drought (Figure 4B). The severe shrinkage in the SWB area observed during the dry winter might have been driven by high water demand, limited winter precipitation, and rapid depletion of surface water reserves due to drought. The SWB area anomalies during the dry pre-monsoon season are relatively less intensive than the dry monsoon and winter seasons, indicating a mean shrinkage of only 2%. Additionally, the impact of consecutive dry seasons remains minimal during the pre-monsoon (Figure 4D).

Overall, the SWB show considerable variation in water area across seasons, climate zones, and drought conditions. During dry seasons and years, the highest shrinkage in surface water area is observed in the Semi-arid, Humid subtropical, and Tropical Wet and Dry climate zones, which might be driven by high water demand, limited winter precipitation, and rapid depletion of surface water reserves. In contrast, the SWB in the Mountain Frost climate zone shows an expansion of water area driven by warming (Figure S3). The impact of drought is most evident during the summer-monsoon and winter seasons, and successive dry conditions in these seasons ramp up water loss by three times compared to individual seasons. A dry summer-monsoon, followed by a wet winter season, is unlikely to restore their long-term mean SWB area.

### Impact of drought severity

**Figure 5:**
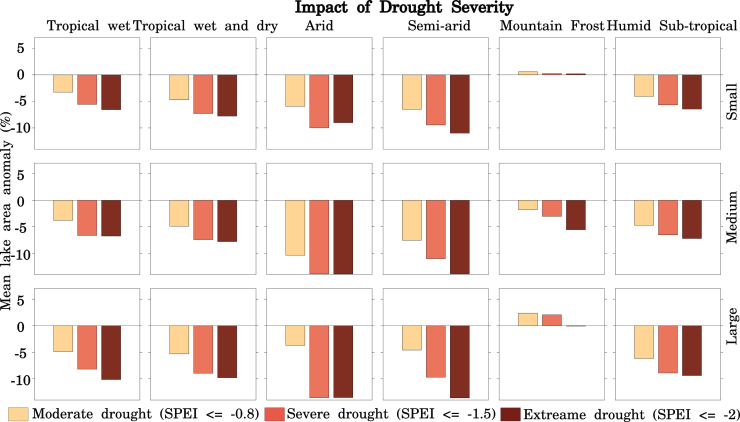
The surface water bodies (SWBs) area anomaly between long-term mean and moderate (SPEI ≤ −0.8), severe (SPEI ≤ −1.5), and extreme (SPEI ≤ −2) drought years from 1990 to 2017 in India The comparison highlights drought impacts on mean annual water area across small, medium, and large SWB within Tropical wet, Tropical wet and dry, Arid, Semi-arid, Mountain Frost, and Humid Subtropical climate zones. The bar plot in the top-to-bottom panels represents the mean water area in small, medium, and large SWB, respectively. We excluded SWBs associated with dense canal networks to avoid anthropogenically influenced SWBs in the Arid climate zone. For more details on anthropogenically influenced SWB, see Figures S4 and S5.

Next, we analyzed the variability in the water area anomaly with increasing drought severity from moderate to extreme conditions in small, medium, and large SWB across climate zones in India. We removed human-influenced SWB with the dense canal network in the Arid region to account for climate-derived drought severity (Figures 5, S4, and S5, S2). Our results show that increased drought severity led to progressive shrinkage, particularly as the size of the SWB increases (Figure 5). The decline in SWB area with increasing SWB size was observed across climate zones, except the Mountain Frost climate zones. Whereas the SWB area is expanding as drought severity increases in the Mountain Frost climate zones. SWB in the Arid and Semi-arid zones is sensitive, exhibiting the largest reductions in water area across all sizes, particularly in medium and large SWB (exceeding 3% decline under extreme drought). This is likely due to limited precipitation, which is highly variable during droughts, and insufficient to compensate for high evapotranspiration, exacerbating shrinkage during drought events.^35^ SWB across all sizes in the Tropical Wet and Humid Subtropical climate zones show a progressive reduction in SWB area with increasing drought severity, with large SWB exhibiting the sharpest decline (up to ∼10%) under extreme drought. These climate zones receive frequent and intense rainfall, maintaining water levels.^36^ During droughts, a sharp drop in precipitation combined with persistent high evapotranspiration significantly reduces surface water availability.^11^ Although the Humid Subtropical climate zone is rich in water resources, the high population density and agricultural needs in this region exacerbate water stress during droughts due to increased anthropogenic withdrawals. The Tropical Wet and Dry zones, characterized by their clear wet and dry seasons, show substantial declines in the SWB area during droughts, particularly in large and shallow SWB that rely heavily on direct rainfall.^37^ In contrast, the small SWB in the Mountain Frost zones shows the expansion of SWB under moderate drought, possibly because of the glacier and snow melting in the Himalayas due to increasing temperature under climate change.^38^^,^^39^ The response glacier and snow melt due to increasing temperature resulted in the rapid emergence of more small glacial SWB surrounding the Himalayan glaciers.^40^ However, these contributions diminish under severe and extreme droughts, where the medium to large SWB begin to show significant reductions.

Overall, the results demonstrate that drought intensification resulted in widespread shrinkage in SWB area with an increase in SWB size in all the climate zones, except the Mountain Frost climate zone. The large SWB experience greater percentage reductions due to their higher exposure and dependence on widespread runoff. Arid and Semi-arid climate zones are most severely affected (>13% decline), followed by Tropical wet, Humid Sub-tropical, and Tropical wet and dry climate zones (∼10% decline) under extreme droughts. However, SWB in the Mountain Frost climate shows expansion during extreme droughts. The small and large SWB in the Mountain Frost climate zone are expanding, but the extent of expansion decreases with increasing drought severity in the medium SWB. These insights are crucial for shaping effective water management strategies as we face future climate change and prolonged droughts.

### Shrinkage rate

**Figure 6:**
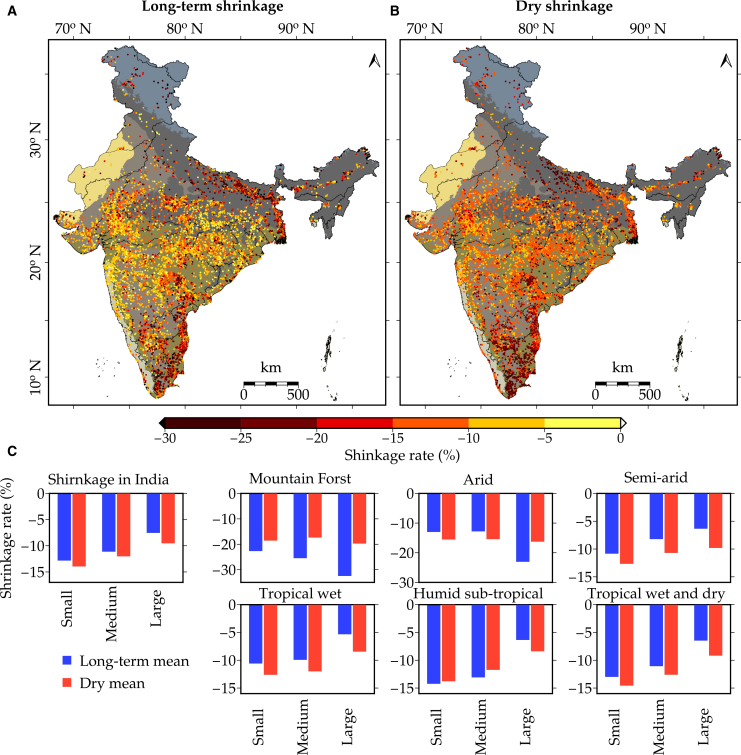
The spatial distribution of surface water bodies’ (SWBs) shrinkage rate (A) The spatial variability in long-term mean shrinkage rate. (B) The spatial variability of the dry period shrinkage rate during the water cycle year in India. The scale bar, ranging from light yellow to dark brown, indicates the higher shrinkage rates. (C) The climate-wise mean shrinkage rate of small, medium, and large SWBs in India. For details on shrinkage duration and period, see Figure S6.

We quantified the mean annual shrinkage rates in the water area for a water year, where the more negative values indicate higher shrinkage and values closer to zero indicate minimal shrinkage (Figure 6). During the long-term mean period, the SWB in the Semi-arid, Tropical wet, and Tropical Wet and Dry climate zones of the central part of India show a relatively lower shrinkage rate (−10% to −5%) than the Humid sub-tropical in the Ganga plain, Mountain Frost in North India, and Tropical Wet and Dry in southern part of India (Figures 6A and 6B). On the other hand, the SWB in southern India and the northern Ganga plains show relatively higher shrinkage rates (−30% to −20%) (Figure 6A). During dry years, the shrinkage rate increased and expanded across all the climate zones in India (Figure 6B). Particularly, SWB in central India exhibits intensified shrinkage rates, ranging from −10% to −5% in normal conditions to −15% to −10% in the dry years (Figure 6B). Additionally, an early shift in the highest-shrinkage period is observed (Figure S6) during the drought years, which further prolongs water-stress conditions in the region.

The shrinkage rates during the dry years and long-term mean show that the small SWB have experienced the highest shrinkage rate, followed by medium and large SWB across climate zones, except the Mountain Frost climate zone in India (Figure 6C). This size-dependent response is evident across most climate zones, reflecting the limited storage capacity and rapid hydrological response of small water bodies. (Figure 6C). In contrast, Mountain Frost climate zones show distinct behavior, where small SWB show relatively less shrinkage than medium and large SWB during normal conditions. However, the shrinkage rate declined further during the dry period in the Mountain Frost climate zone.

Overall, the shrinkage rates highlight that drought conditions systematically intensify the water loss have increased (by 2%–3%) across India, with the magnitude of shrinkage controlled by the SWB size and hydro climatic conditions Specifically, the small SWB have experienced a relatively higher shrinkage rate (−14%) than medium (−12%) and large (−10%) SWB during the dry period.

## Discussion

### Implications of the study

We assessed the impact of annual and seasonal droughts on water areas in the respective periods. During the dry years, most of the SWB across the climate zone show shrinkage in water area, while the SWB in the Mountain Frost climate zone exhibits persistent expansion in water area. Our findings reveal that successive dry conditions during the summer-monsoon and winter seasons in a water year led to a shrinkage in water area nearly three times that during individual season droughts. Subsequently, the impact of increasing drought intensity on the SWB area accelerates shrinkage, highlighting that it considerably affects all SWB sizes in India. However, large SWB exhibit the highest decline in water area as the drought severity increases, likely due to their exposure to evaporation and dependency on runoff, where there is a decline in runoff and an increase in evaporation. In contrast, small SWBs shrink faster relative to their size, which might be due to shallow depth and limited inflow, making them more vulnerable to short-term climate stress.

The observed spatial variability in SWB area is closely associated with land-use and cover (LULC) changes and regional climatic contrasts (Figures S7 and S8). In the Ganga plains, cropland expansion (Figures S8A and S8B) combined with declining precipitation (Figure S7B) and rising temperatures (Figure S7A) has resulted in a substantial reduction of the SWB area exceeding 60% (Figures 2A and 2D). In addition, intensive groundwater extraction for irrigation has significantly depleted groundwater storage in the region.^41^^,^^42^ Such unregulated groundwater abstraction lowers water tables, disrupts groundwater-SWB interactions, and accelerates SWB shrinkage.^43^ Similarly, SWBs in the South Indian sub-basins (Godavari, Krishna, Cauvery, and Penna) have also experienced pronounced declines (40%–60%), despite increasing precipitation and relatively stable temperatures. The contrasting response suggests the role of anthropogenic activities, including cropland expansion, increasing impervious surfaces (Figure S8), and unsustainable water management. In contrast, in the Mountain Frost climate zones, more than 80% of the SWB shows an increasing trend, primarily influenced by approximately 20,000 km^2^ of glacier and snow cover loss. The snow cover loss is driven by a significant increase in temperature (>0.4 °C/decade) and declining precipitation (4mm/year) (Figure S3). Rapid warming and reduced precipitation drive snow cover loss, making meltwater the dominant source of SWB expansion in the Mountain Frost climate zone (Figure S7).

Similarly, more than 40% of SWB in western-flowing (i.e., Narmada, Mahi, Sabarmati) and eastern-flowing Mahanadi Basin show a significant increasing trend, despite rising temperatures, driven by increased precipitation (Figures 2A and 2C). The analysis of spatial patterns in SWB area anomalies during drought years and seasons further indicates that declining water areas are closely associated with increasing temperatures and reduced precipitation, which together intensify drought severity. Additionally, excessive use of surface water and groundwater extraction for irrigation during drought periods further exacerbates long-term stress on SWB.^44^^,^^45^

Addressing the water area anomalies, shrinkage rate, and shrinkage duration under increasing drought intensities provides valuable information in understanding the vulnerability of the SWB. Additionally, these matrices are critical for informing drought preparedness and water scarcity mitigation strategies, enabling policymakers and local stakeholders to implement actions on time. The highly sensitive SWB to droughts, particularly in arid, semi-arid, humid subtropical, and tropical wet and dry regions, has important implications for water policy and drought management. The drought impact on small, medium, and large SWB categories suggests that small SWB (<1 km^2^) require more attention due to their rapid depletion, while large SWBs demand basin-scale coordination to reduce the losses during prolonged droughts. Beyond hydrological impacts, SWB shrinkage during droughts threatens aquatic habitats and shoreline vegetation and intensifies ecological stress by increasing water temperature and salinity, concentrating nutrients, and promoting algal blooms that deplete oxygen, degrade habitats, and cause biodiversity loss.^46^^,^^47^ Additionally, groundwater governance plays a crucial role in maintaining SWB^43^ as excessive pumping can transform SWB from recharge zones into groundwater sinks and vice versa, thereby accelerating the impacts of drought. Integrated surface-groundwater management policies are therefore essential for sustaining both surface and subsurface water resources during droughts. Moreover, protecting SWB from encroachment, preserving natural drainage pathways, and restricting land-use change within optimal catchment buffer zones improve rainfall capture, slow down water loss during droughts, and sustain storage. In river-connected systems, maintaining minimum environmental flows can further prolong downstream SWB persistence during droughts.

In particular, identifying SWB affected by droughts in the semi-arid regions of central and south India, and the tropical sub-humid region of the Ganga plains, can be used to restore and conserve water resources in areas with high recharge potential.^48^ Conserving SWB over highly permeable or unconfined aquifers within the tropical sub-humid climate zone can substantially enhance natural groundwater infiltration.^49^^,^^50^^,^^51^ Additionally, the basaltic aquifer in the semi-arid region of central India, particularly in the Deccan basalt region, is more suitable for localized groundwater recharge^52^ that can help sustain baseflows during dry periods. These insights align with the national water initiatives such as the National Lake Conservation Plan (NLCP), Jal Jeevan Mission (JJM), and Jal Shakti Abhiyan (JSA), which focus on water conservation, rural water supply, sustainable water management, and enhancing surface-groundwater interaction. Our research underscores the need to incorporate lake dynamics into long-term, basin-scale hydrological planning, including lake interlinking, rainwater harvesting, and multi-source water budgeting. Additionally, our findings indicate that medium-sized (1–10 km^2^) and large-sized (10–100 km^2^) SWB are more drought-resilient than smaller water bodies, as they exhibit lower shrinkage rates during dry conditions. Consequently, future SWB creation and restoration efforts should prioritize the development of medium-large-sized water bodies to enhance long-term drought resilience. The correlation between monthly water area and the SPEI-1 shows a 3-month lag, indicating a delayed response of the hydrological system to runoff. The droughts reduce precipitation, lower runoff and inflow into the SWB, and increase evaporative losses from the SWB. Droughts have led to an earlier shift in peak shrinkage, highlighting the increased sensitivity of SWB (Figure S6). Moreover, the observed disproportionate shrinkage of SWB under extreme and successive droughts indicates non-linear responses to increasing drought severity, consistent with findings of meteorological-hydrological response studies.^53^^,^^54^

### Limitations of the study

Despite a comprehensive assessment of drought impacts on SWB, several potential limitations remain. This analysis is solely based on the SWB area and does not provide insights into actual volume changes in SWB. The absence of altimetry water-level data further constrains volumetric variability in a large number of SWB.^55^^,^^56^ In addition, the SWB area may contain uncertainties arising from surface water classification using Landsat imagery. The classification accuracy is primarily affected by cloud cover, shadows from hills or clouds, aquatic vegetation, and mixed pixels at the edges, particularly for SWB with steep banks, shallow depth, and small sizes.^13^ The major limitation of this study is that it attributes SWB shrinkage primarily to drought severity, without explicitly incorporating irrigation withdrawals. Particularly, the absence of irrigation withdrawal data in intensive irrigated regions may overestimate the climate contribution. Nevertheless, the strong correlation between annual water area and SPEI-12 (r = 0.72) suggests that climate variability plays a dominant role in governing the long-term SWB variability. Moreover, we did not consider water loss due to evaporation or human interventions, such as dams, canals, and groundwater extraction, which may significantly influence surface water dynamics.^57^ The long-term, unsustainable irrigation of surface water withdrawals and groundwater pumping, and land-use change often lead to persistent negative annual anomalies. Conversely, controlled inflows from canals or rivers can sustain or increase SWB anomalies that nullify climatic drivers. In addition, the lack of frequent groundwater in the proximity of SWB also constrains understanding of surface water and groundwater interactions.

Future research can incorporate surface water and ocean topography (SWOT) Satellite Mission surface water elevation,^58^^,^^59^ coupling high-resolution satellite-based data for SWB with lake/reservoir models^60^^,^^61^ alongside *in situ* monitoring, improving estimates of storage changes, especially for small or regulated SWB. Moreover, integrating lake area changes with ecological indicators and water-quality parameters would enhance the understanding of the effects on habitat availability and ecosystem functioning.^62^ Future work should integrate observational or hydrological model irrigation withdrawals and groundwater datasets to distinguish between climatic and anthropogenic drivers of SWB variability. Furthermore, citizen science and *in situ* observations could support ground-truthing of small or ephemeral SWBs. Finally, scenario-based modeling of lake responses to future climate extremes can inform targeted strategies for lake restoration, drought management, and water sustainability.

We investigated the impacts of droughts on the SWBs across India using long-term satellite-derived water area data from 1990 to 2017. Based on the findings of this study, the following conclusions can be drawn.

1. The annual and seasonal droughts have remarkably affected the SWB area in semi-arid, Tropical-wet, and dry climate zones, with summer-monsoon droughts initially affecting the SWB area in central India and subsequently expanding to southern regions during the winter season.
2. The dry summer-monsoon season, and even subsequent wet seasons in the same year, are unlikely to revive the water in SWB. Combined summer-monsoon and winter droughts result in a decline in water area nearly three times greater than monsoon droughts alone, highlighting the compounding impact of back-to-back dry seasons.
3. Increasing drought severity reduces the mean annual SWB area across climate zones, with large SWB showing the highest negative anomaly (ranging from −14% to −8.14%), except in the Arid and Mountain Frost zones, where the small SWB show more resilience.
4. Shrinkage rates are highest, particularly in Semi-arid central India and the Ganga basin sub-humid regions. In contrast, SWB in the Mountain Frost zone remain the least affected, indicating regional and size-based disparities in droughts.

## Resource availability

### Lead contact

Further information and requests for resources should be directed to and will be fulfilled by the lead contact, Vimal Mishra (vmishra@iitgn.ac.in). This study did not generate any new and unique codes.

### Materials availability

This study did not generate new unique reagents.

### Data and code availability

• The descriptions of data are listed in the key resources table.
• This study did not generate any new and unique codes.
• All other datasets that support the findings of this study are available at https://doi.org/10.5281/zenodo.17048172. Any other information can be provided by the corresponding author upon reasonable request.

## Acknowledgments

Authors acknowledge the data-providing agencies. This study was supported by funding from the Major Research and Development Program (MRDP) in Hydroclimatic Extremes from the 10.13039/501100001409Department of Science and Technology (DST), Ministry of Science and Technology, India (Grant: MRDP43265).

## Author contributions

V.M. designed the study. M.N. performed the analysis and wrote the first draft. Both authors have contributed to the writing, review, and editing.

## Declaration of interests

The authors declare no competing interests.

## STAR★Methods

### Key resources table

**Table:** 

REAGENT or RESOURCE	SOURCE	IDENTIFIER
**Deposited data**		

Monthly SWB data	Donchyts et al.^13^ and Global lake evaporation volume (GLEV) dataset^21^	figshare: https://doi.org/10.6084/m9.figshare.20359860 Zenodo: https://doi.org/10.5281/zenodo.4646621
SPEI data	Chuphal et al.^62^	Zenodo: https://zenodo.org/records/8280551
HydroBasins level-8 and 5 data	Hydrosheds website	https://www.hydrosheds.org/products/hydrobasins
Köppen–Geiger climate zone classification data	GloH2O website^63^	https://www.gloh2o.org/koppen/
LULC dataset	Zhang et al.^64^	https://doi.org/10.5194/essd-16-1353-2024
Snow cover data	MODIS satellite via Google Earth Engine	https://developers.google.com/earth-engine/datasets/catalog/MODIS_061_MOD10A2

**Software & Algorithms**		

Source codes and datasets	Authors	Zenodo: https://doi.org/10.5281/zenodo.17048172

### Method details

#### Data

We used the recently published Donchyts et al. (2022),^13^ extensively validated high-resolution surface water dynamics of small (0.1–1 km^2^) and medium (1–10 km^2^) sized global monthly water surface area datasets for the 1985–2020 period.^13^ The monthly water surface area dataset is derived from NASA Landsat-4, 5, 7, 8 and ESA Copernicus Sentinel-2 images using a water classification algorithm in the Google Earth Engine platform.^13^ In addition, we obtained the monthly surface water area datasets from the global lake evaporation volume (GLEV), which includes small, medium, and large SWB (more than 10 km^2^).^21^ The GLEV dataset was reconstructed based on a combination of the dynamic Landsat-based global surface water dataset (GSWD) and static HydroLAKES shapefiles from 1985 to 2018. These datasets have demonstrated strong performance in capturing global SWB water area dynamics and provide reliable estimates of relative changes in SWB area. However, due to limited satellite observations, several SWB in these datasets have continuous missing values. We have selected the SWB with long-term continuous observations. After combining these two datasets, we selected 10,476 SWB with continuous long-term water surface area for the 1990–2017 period. These two datasets provide small, medium, and large sized SWB water surface area time series that spread throughout India (Figure 1; Table 1). Additionally, we used the long-term (1901–2021) high-resolution (0.05°) Drought atlas datasets from Chuphal et al. (2024),^62^ which provide the 1-month, 4-month, and 12-month Standardised Precipitation and Evapotranspiration Index (SPEI) datasets.^65^ The SPEI datasets were prepared using high-resolution bias-corrected precipitation and minimum and maximum temperatures from the India Meteorological Department (IMD) and other sources (CHRIPS) Funk et al. (2015)^66^ for India. We used the HydroBASINS Level-8 dataset to derive precipitation, temperature, and SPEI dataset for each sub-basin by taking the mean of all the gridded data (5 km) within a particular basin to study the SWB area variability under drought conditions. We used the Köppen–Geiger climate zone classification data^63^ to examine drought response on SWB across the different climate zones.

**Table 1:** The SWB classification criteria and their count

SWB categories	Criteria	Number of SWB
Small	1.1–1 km^2^	8636
Medium	1–10 km^2^	1612
Large	>10 km^2^	228

#### Long-term trend analysis

We performed the non-parametric Trend-Free Pre-Whitening Mann–Kendall (TFPW-MK) test to assess long-term (1990–2017) trends in the SWB area^67^^,^^68^^,^^69^ (Additional details can be found in Supplement text S1). From these analyses, we obtained the *p*-value, significance level (α), and Sen’s slope. The trends are considered statistically significant at the 95% confidence Interval (α = 0.95 and *p*-value <0.05) (Refer Supplementary Methods S1 for additional information). We then multiplied Sen’s slope with the duration (number of years in the 1990–2017 period) and estimated the total change (%) in SWB area to compare relative changes across all size categories, as expressed in the equation below:

(Equation 1) $$Total\;change\;\left(\%\right)=\left(\frac{Sen^{\prime }s\;slope}{\mathrm{Max}\;lake\;area}\right)*100*no\;of\;years.$$

#### Spatiotemporal variability of water area during drought

We considered the monthly SWB area in a water year (June-May) from 1990 to 2017 to evaluate the spatiotemporal variability in monthly, inter-annual, and seasonal [i.e., summer-monsoon (June-September), winter (November-February), and pre-monsoon (March-May)] water area. We estimated the annual mean water area by taking a mean of the monthly SWB area for each water year from 1990 to 2017, which allows us to examine the inter-annual variations in the SWB area. Firstly, we considered 1-month SPEI (SPEI-1), 4-month SPEI (SPEI-4), and 12-month SPEI (SPEI-12), and estimated correlations between monthly, seasonal (Monsoon, Winter, and Pre-monsoon), and annual mean water area at the corresponding time step. After checking their agreements at respective time steps, we selected the highly correlated SPEI-4 to examine the impacts of seasonal droughts and SPEI-12 to assess the annual impacts of drought severity on SWB. We categorised the droughts (SPEI<−0.8) and wet (SPEI>0.8) according to Chuphal et al.^62^ Next, we analyzed the annual water area anomalies under moderate (−1.5 ≤ SPEI ≤ −0.8), severe (−2 ≤ SPEI ≤ −1.5), and extreme (SPEI ≤ −2) drought conditions (Figure 5), as well as their effects on small, medium, and large SWB across climate zones in India.

**Figure 7:**
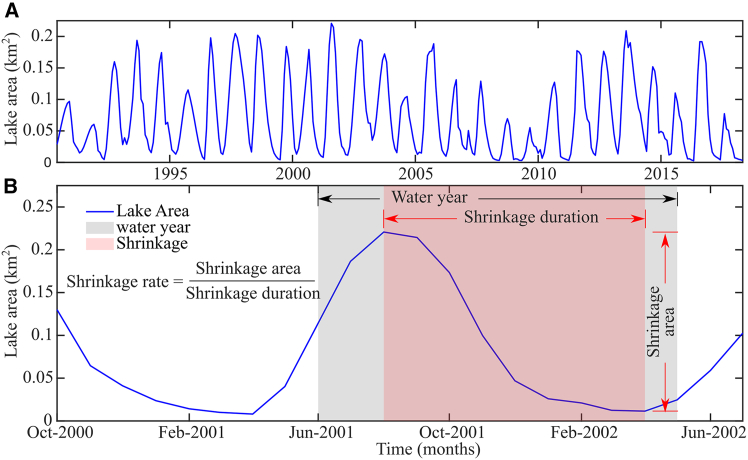
The schematic representation of the shrinkage rate and duration estimation in a surface water body (A) The time series of water body surface area from 1990 to 2017 (location 25.00833, 80.9493). (B) A schematic representation of the surface water area, shrinkage duration, and shrinkage rate in a water year. For the details on shrinkage duration and periods, see Figure S6.

We estimated the shrinkage rate of the SWB area by dividing the change in water area between the peak (maximum) and subsequent trough (minimum) with the shrinkage duration (number of months between peak and trough) in each water year (June to May, Figure 7). Then, we analyzed the shrinkage rates and duration for the dry periods to evaluate the impact of the drought on the shrinkage of the SWB area. Overall, the multi-temporal (i.e., monthly, seasonal, annual) analysis of the SWB area allows us to identify the observed changes in the water area and assess the impact of drought on surface water dynamics.

### Quantification and statistical analysis

We used the Trend-Free Pre-Whitening Mann-Kendall test (TFPW-MK) to estimate trends and the Wilcoxon rank-sum test to evaluate significant changes between two groups. We used MATLAB 2023b for all statistical analyses in this study; QGIS for spatial and geographical analyses; and Generic Mapping Tools (GMT) to prepare figures.
