# Supplementary material for: Drought driven shrinkage of surface water bodies in India
Source: iScience. 2026 Jul 13;29(8):116737. doi: 10.1016/j.isci.2026.116737 (PMC13382329; doi:10.1016/j.isci.2026.116737)
Supplement: Document S1. Figures S1–S9, Table S1, and Methods S1 and S2 [file mmc1.pdf]

iScience, Volume *29*

Supplemental information

Drought driven shrinkage of surface water bodies in India

M. Niranjannaik and Vimal Mishra

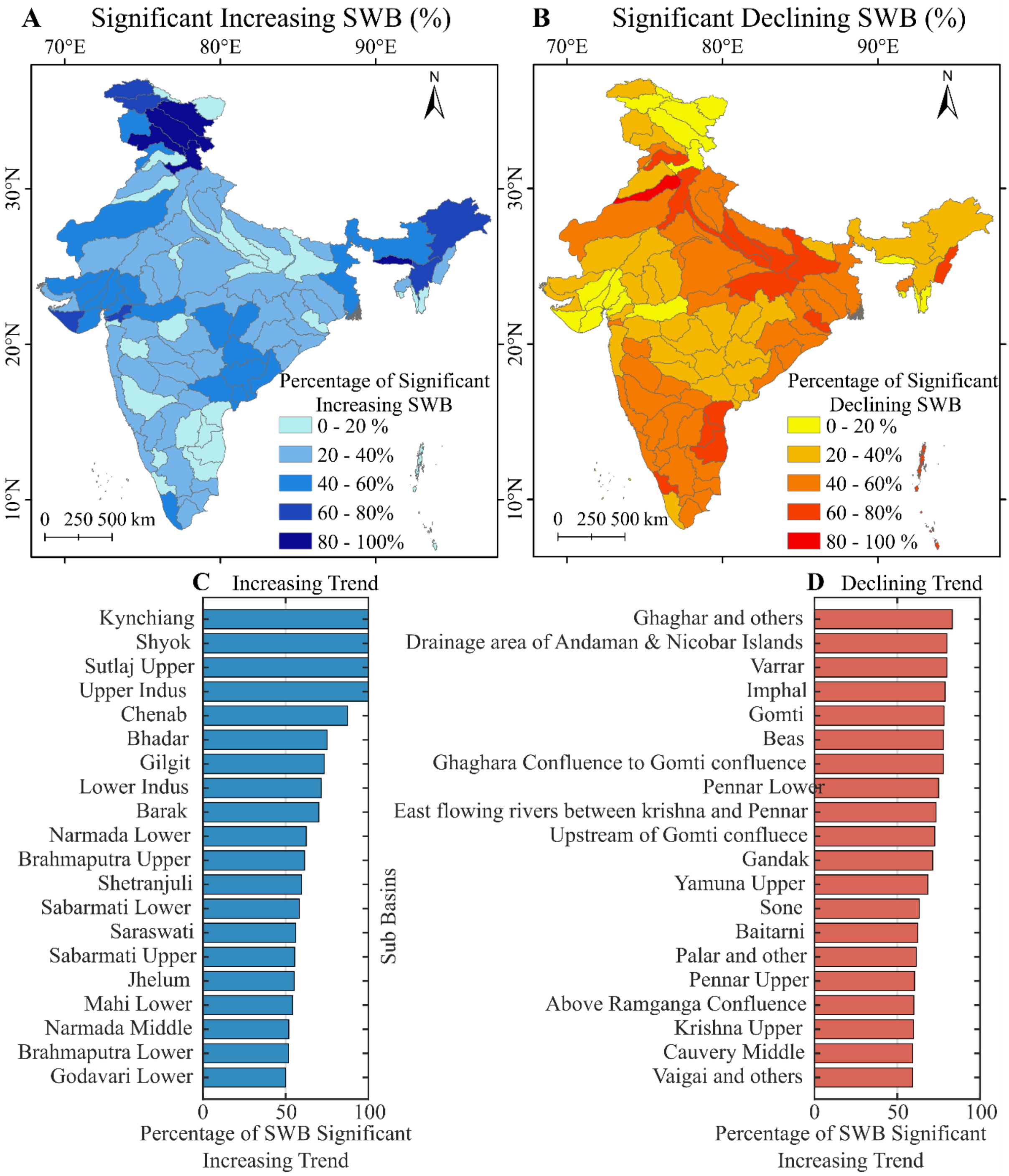

Figure S1. The spatial distribution of the percentage of SWB with Significant trends in respective subbasins. (A) Subbasin-wise percentage SWB with a significantly increasing trend. (B) Basin-wise percentage SWB with significant declining trends in each subbasin. (C) The top 20 subbasins with the percentage of SWB having significant increasing trends are shown with blue bars. (D) The top 20 subbasins with the percentage of SWB having significant declining trends are shown with red horizontal bars. Related to Figure 2.

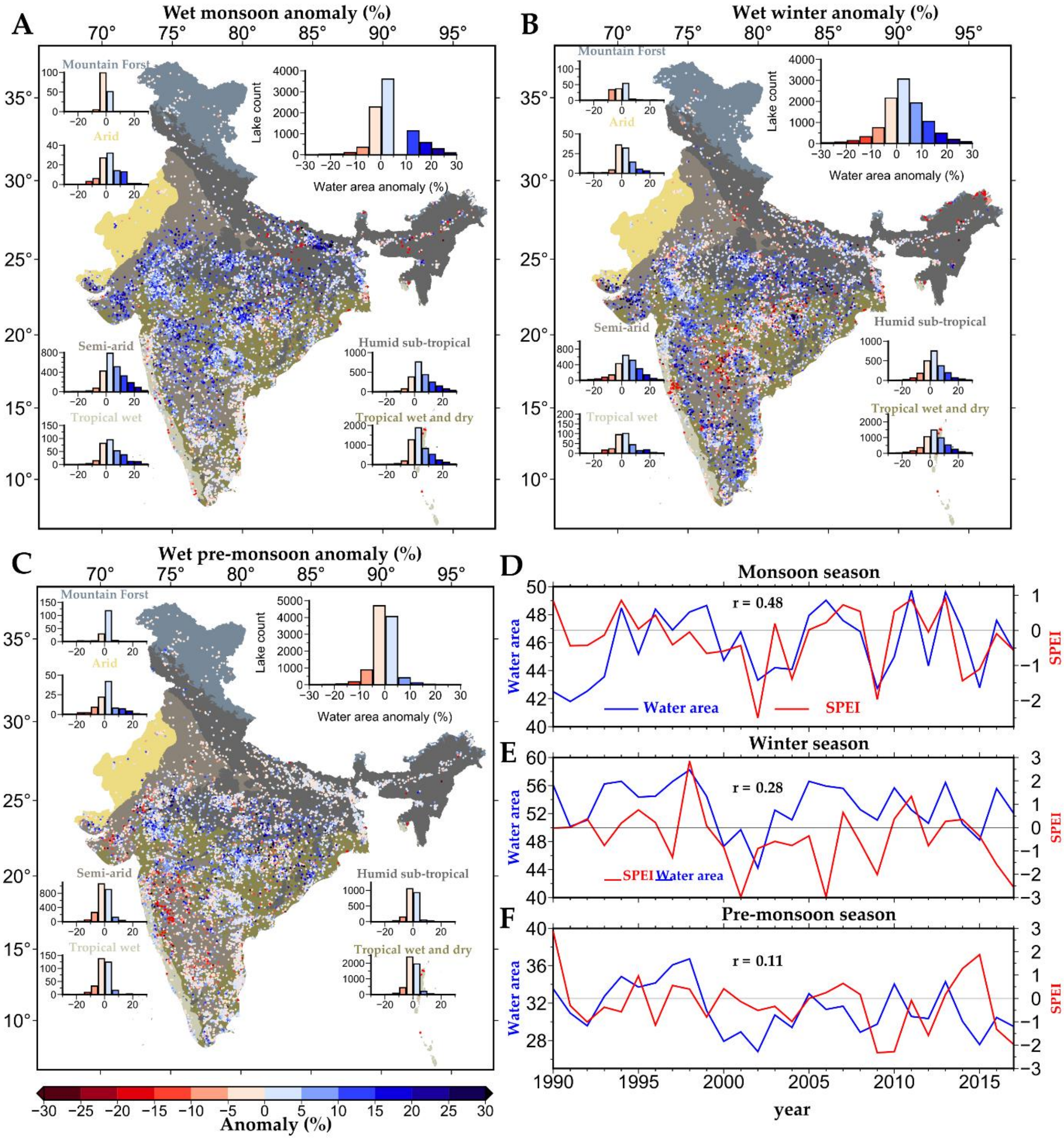

Figure S2. The spatial map of seasonal variability in surface waterbodies (SWB) area anomaly (%). (A) Wet monsoon season water area anomaly, (B) Wet winter season water area anomaly, and (C) Pre-monsoon season water area anomaly, with the histogram of SWB water area anomalies with a range of 5 % for each climate zone over India. The negative anomalies are shown in red, and the positive anomalies are shown in blue. The red SWB circles and bars show negative anomalies, and the blue ones show the positive anomalies. (D-F) Shows the comparison between the mean water area (%) (shown with blue line) and standardised Precipitation Evapotranspiration Index during the monsoon, winter, and pre-monsoon seasons, respectively. Related to Figure 4.

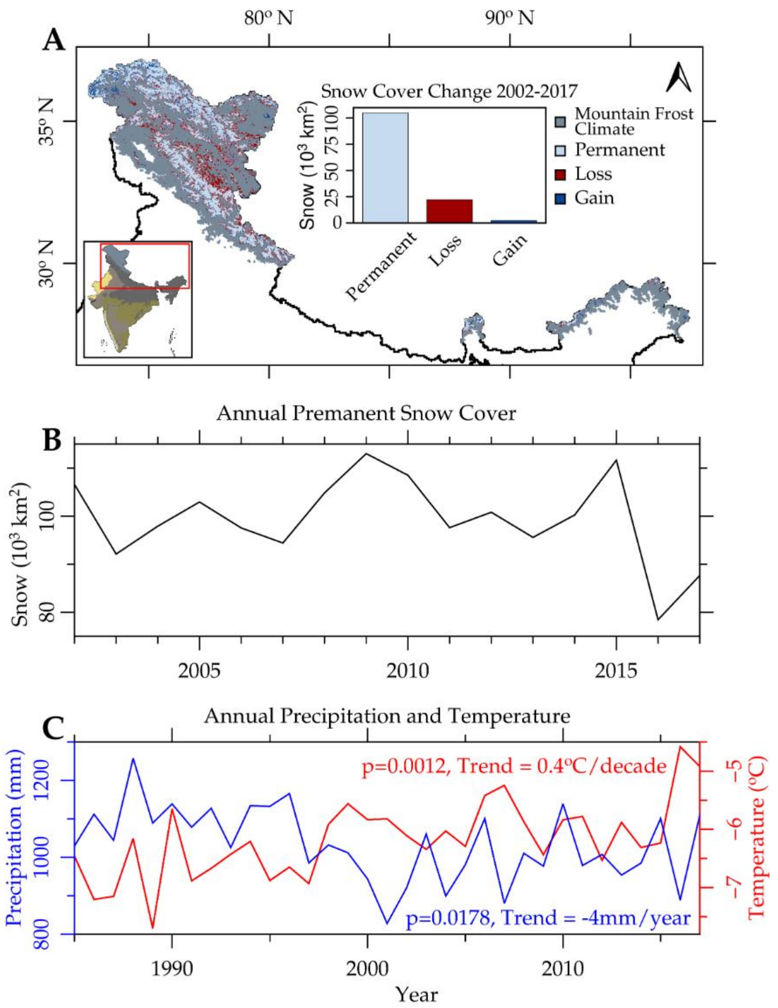

Figure S3. Spatiotemporal variability in snow cover and climate variables. Satellite-derived annual permanent snow cover area (km^2) changes from 2002 to 2017 and temporal variability in hydroclimate variables. (A) The spatial map of permanent snow cover, loss, and gain from 2002 to 2017. The permanent snow cover, snow gain, and snow loss regions are represented with the light blue, dark blue, and dark red, respectively. (B) Time series of annual permanent snow cover area. In addition, (C) illustrates the temporal variability in hydroclimatic parameters (i.e., precipitation and temperature), along with the p-value and trends. The blue and red lines indicate the precipitation and temperature, respectively. Related to Figure 4.

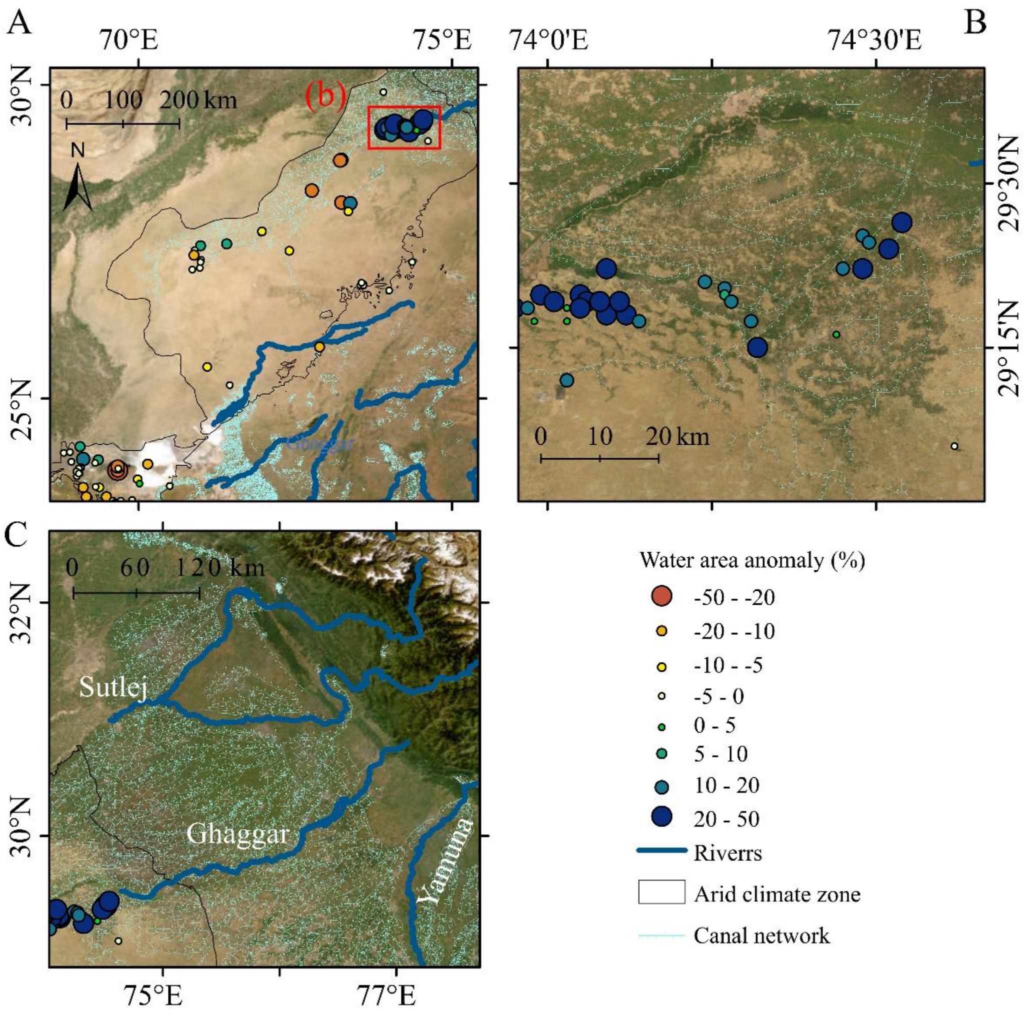

Figure S4. The increasing SWB water area anomaly during pre-monsoon drought and its causes. (A) The spatial distribution of the SWB area anomaly (%) during drought in the Arid climate zone of India shows a cluster of 28 SWB with high shrinkage in the water area anomaly in the Northeastern region of the arid climate zone. The dark blue and dark brown circles indicate highly positive anomaly negative anomalies, respectively. (B) The cluster of 28 SWB with high shrinkage during drought is situated in the densely connected canal network (light blue) region. (C) The river (dark blue lines) and the dense canal network are connected to the cluster of 28 SWB. Related to Figure 5.

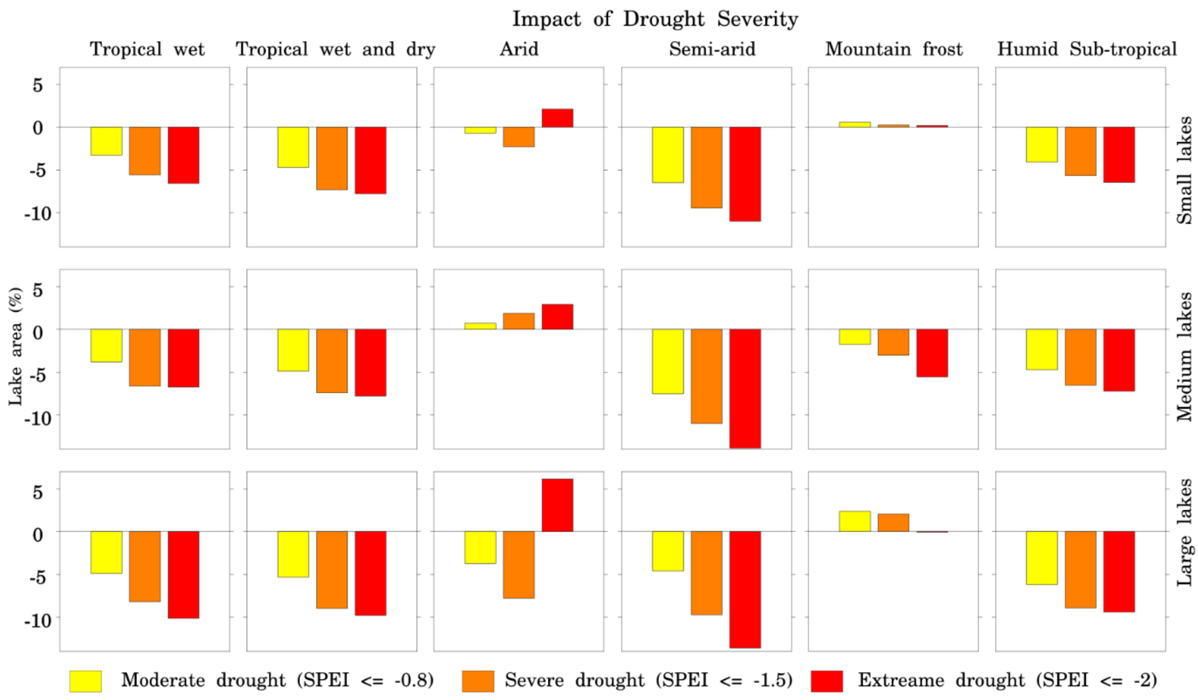

Figure S5. The drought severity impact on SWB. The SWB area anomaly between long-term mean and moderate (yellow), severe (orange), and extreme (red) drought years in India from 1990 to 2017. The comparison highlights drought impacts on mean annual water area anomaly during moderate, severe, and extreme droughts across small, medium, and large SWB within Tropical wet, Tropical wet and dry, Arid, Semi-arid, Mountain Frost, and Humid Subtropical climate zones, respectively. The bar plot in the top-to-bottom panels represents the mean water area for small, medium, and large SWB, respectively. Related to Figure 5.

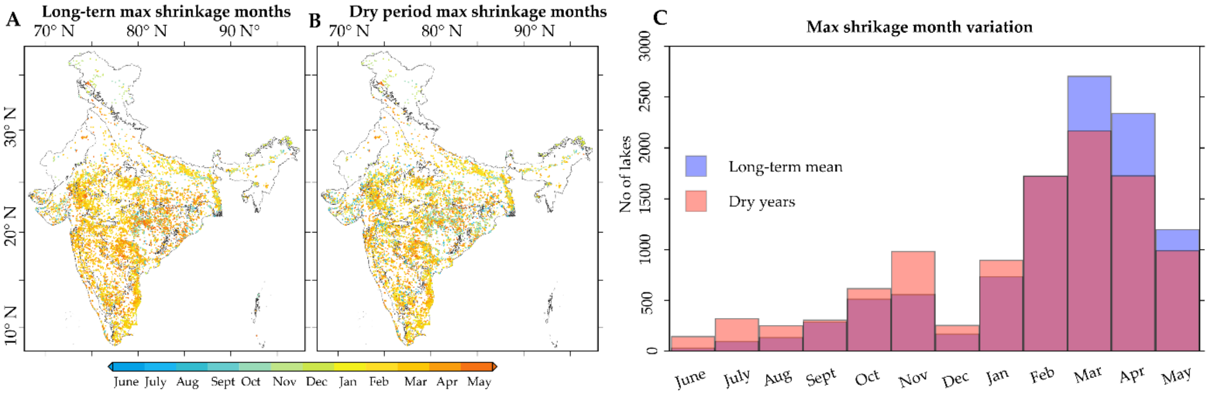

Figure S6. The spatial map of maximum (max) lake water area shrinkage (%). (A) long-term mean max water area shrinkage and (B) dry years max water area shrinkage. (C) shows the bar plot of maximum lake area shrinkage months during the long-term mean (blue bars) and dry years (red bars). Related to Figure 7.

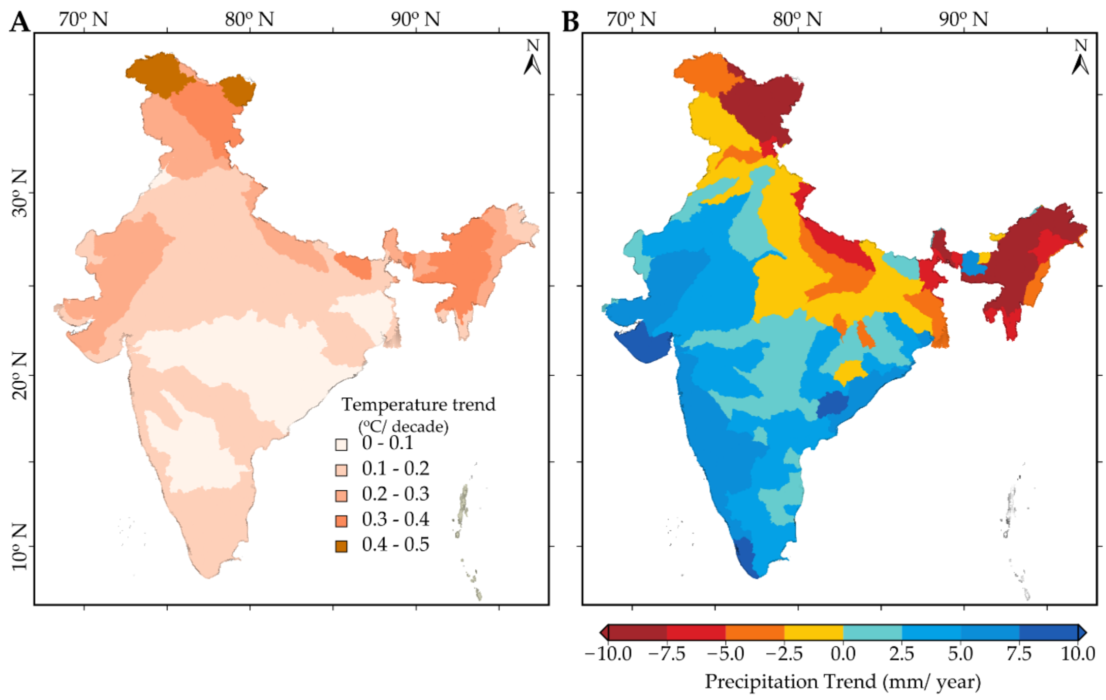

Figure S7. The long-term (1990-2017) spatiotemporal trends in hydroclimatic parameters. (A) Subbasin-wise spatial map of long-term trends in temperature (ºC/ decade). (B) Subbasin-wise spatial map of long-term trends in precipitation in mm/year, at the sub-basin scale. The precipitation shades from light blue to dark blue show high increasing trends, and from yellow to dark red indicate highly declining trends. Related to STAR methods.

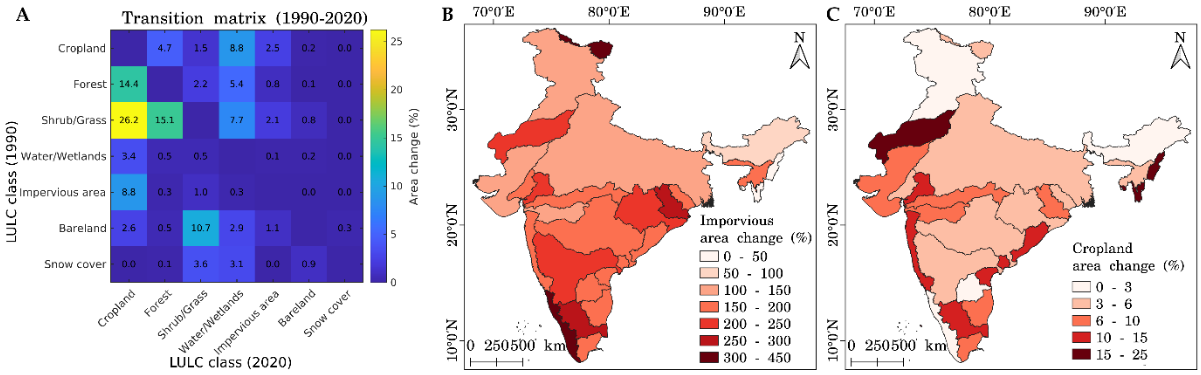

Figure S8. Comparison of changes in SWB water area with the land use and land cover (LULC). (A) The LULC transition matrix for India shows the spatio-temporal conversion of land cover classes from 1990 (rows) to 2020 (columns) using the GLC_FCS30D datasets. Each cell in the matrix represents a change in area that transitioned from a specific LULC class in 1990 to another class in 2020. Whereas the diagonal elements maintain persistent coverage, the percentage change in area remains zero. For example, the cell in row 3, column 1, with an area of 26.2%, represents the transition from the shrub/grassland to crop land class from 1990 to 2020. The transition matrix, with blue indicating the lowest conversion and yellow the highest, is shown as percentages. (B) Basin-wise spatial variability in the percentage change in impervious area. (C) Basin-wise spatial variability in the percentage change in cropland at the basin scale from 1990 to 2020. Related to STAR methods.

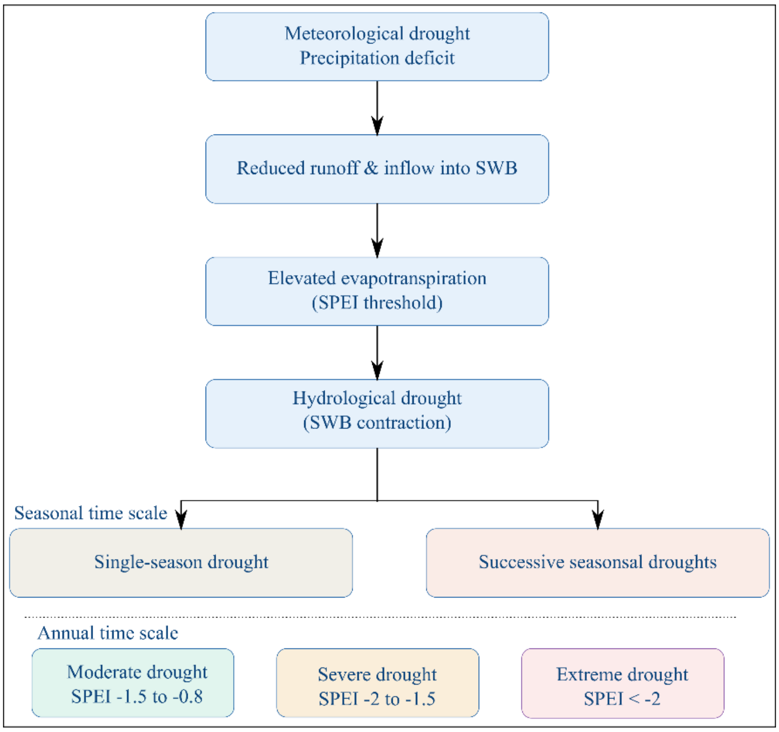

Figure S9: The conceptual framework illustrating the drought propagation from meteorological to surface water bodies (SWB). The drought impacts in the SWB area are evaluated at the seasonal (monsoon, winter, pre-monsoon) scale, and drought severity is assessed at the annual scale (i.e., moderate, severe, and extreme droughts).

Table S1: Wilcoxon rank-sum test results comparison of single-season and successive droughts SWB water area change. The table reports p-values, median differences (%), effect sizes, and 95% confidence intervals (CI). Negative median differences indicate greater shrinkage under successive drought conditions.

Successive drought comparison with	P-value	Median difference	Effective size	CI lower	CI upper
Monsoon	0.0002	-4.35	0.16	-4.65	-4.03
Winter	0.00002	-5.25	0.28	-5.54	-4.94

Methods S1: Trend-Free Pre-Whitening Mann-Kendall test (TFPW-MK) (related to STAR Methods)

The Trend-Free Pre-Whitening (TFPW)[1] method removes the autocorrelation without distorting the underlying trends in the time series. After the Trend-Free Pre-Whitening, we applied the Mann-Kendall (MK)[2] test to detect trends without autocorrelation.

The Trend-Free Pre-Whitening (TFPW) test procedure is shown as follows:

Step-1: The linear trend is estimated using Sen's slope.

$$X_t = \beta t + \epsilon_t$$

Where X_t is the observed water area at time t of a SWB, β the trend slope, ϵ_t is the residual at time t.

Step-2: The estimated trend is removed

$$Y_t = X_t - \beta t$$

Where Y_t is the detrended time series.

Step-3: Then removed the autocorrelation from a detrended time series Y_t by estimating the lag-1 autocorrelation coefficient (r_1).

$$r_1 = \frac{\sum_{i=1}^{n}(Y_t - \bar{Y})(Y_{t-1} - \bar{Y})}{\sum_{i=1}^{n}(Y_t - \bar{Y})^2}$$

$$Y'_t = Y_t - r_1 Y_{t-1}$$

Where r_1 is the lag-1 autocorrelation coefficient and $\bar{Y}$ is the mean.

Step-4: We then reintroduce the trend into the autocorrelation-removed time series Y'_t.

$$X'_t = Y'_t + \beta t$$

Mann-Kendall (MK) test statistics details:

We performed trend analysis of the lake water area to assess long-term (1990-2017) changes. We first deseasonalised the time series by removing the monthly climatological mean from each corresponding month and estimated the trend by employing the Mann-Kendall (MK) test[2,3]. The MK is a non-parametric method that assesses monotonic trends in water area. The

MK test computes whether the trend is significant or non-significant based on the test statistic S of strength and direction, the Z-score of S.

$$S = \sum_{i=1}^{n-1} \sum_{j=i+1}^{n} Sign(x_j - x_i)$$

$$Sign(x_j - x_i) = \begin{cases} +1 & if\ x_j > x_i \\ 0 & if\ x_j = x_i \\ -1 & if\ x_j < x_i \end{cases}$$

Where x_i and x_j are data values in the time series at the corresponding i and j time step, respectively, and $Sign$ is a sign function. Then, the test statistics Z-Score is computed using the test statistics S. The p-value obtained using the test statistics Z-Score assuming a normal distribution at a significance level (α) of 0.05 or 95% confidence level; if the p-value < 0.05, the trend is considered statistically significant and vice-versa.

$$Z = \frac{S}{\sqrt{var(S)}}$$

Additionally, we estimated Sen's slope to determine the rate of change in water area over time; a positive slope indicates increasing water availability, while a negative slope indicates a decline.

$$Sen'slope = \frac{x_j - x_i}{t_j - t_i}, for\ 1 \leq i < j \leq n$$

$$Change\ (\%) = \left(\frac{Sen's\ slope}{Max\ lake\ area}\right) * 100 * no\ of\ years$$

Methods S2: Land use land cover (LULC) change (related to STAR Methods)

We have utilised the Global 30 m land-cover dynamics monitoring datasets (GLC_FCS30D) from 1985 to 2022[4]. Using the GLC_FCS30D data, we conducted a land use land cover change (LULC) analysis from 1990 to 2020 and estimated the transition matrices within the

SWB boundary and their surrounding 500 m buffer zones. The results show that a transition from shrubland to cropland (26%) is the dominant class from 1990 to 2020, indicating a substantial expansion of agricultural areas near the SWB. Followed by transitions from scrubland to forest (15%) and from forest to cropland (14%), highlighting notable land-cover dynamics within the vicinity of SWB. The observed expansion in cropland and changes in vegetation cover around SWB can increase water demand and reduce natural buffering capacity during droughts. Such land-use transitions likely intensify water stress by increasing withdrawals and altering runoff and infiltration processes, which exacerbates the decline in the SWB area during drought periods.

References

1. Yue, S., and Wang, C.Y. (2002). Applicability of prewhitening to eliminate the influence of serial correlation on the Mann-Kendall test. Water Resources Research *38*, 4–7. https://doi.org/10.1029/2001WR000861.

2. Mann, H.B. (1945). Nonparametric Tests Against Trend. Econometrica *13*, 245–259. https://doi.org/10.2307/1907187.

3. Anghileri, D., Pianosi, F., and Soncini-Sessa, R. (2014). Trend detection in seasonal data: from hydrology to water resources. Journal of Hydrology *511*, 171–179. https://doi.org/10.1016/j.jhydrol.2014.01.022.

4. Zhang, X., Zhao, T., Xu, H., Liu, W., Wang, J., Chen, X., and Liu, L. (2024). GLC_FCS30D: the first global 30 m land-cover dynamics monitoring product with a fine classification system for the period from 1985 to 2022 generated using dense-time-series Landsat imagery and the continuous change-detection method. Earth Syst. Sci. Data *16*, 1353–1381. https://doi.org/10.5194/essd-16-1353-2024.
